# Changes in renal function over time in outpatients with eGFR ≥ 30 mL/min/1.73 m^2^: implication for timing of renal function testing before contrast-enhanced CT imaging

**DOI:** 10.1007/s11604-023-01425-y

**Published:** 2023-04-11

**Authors:** Yoshiki Kuwatsuru, Takahiro Hirano, Ryozo Wakabayashi, Juliana Yumi Ishisaki, Hideaki Sokooshi, Ryohei Kuwatsuru

**Affiliations:** 1 Department of Radiology, Graduate School of Medicine, Juntendo University, 2-1-1 Hongo, Bunkyo-ku, Tokyo, 113-8421 Japan; 2 Department of Real-World Evidence and Data Assessment, Graduate School of Medicine, Juntendo University, 2-1-1 Hongo, Bunkyo-ku, Tokyo, 113-8421 Japan; 3 Clinical Study Support Inc., 2F Daiei Bldg., 1-11-20 Nishiki, Naka-ku, Nagoya, 460-0003 Japan

**Keywords:** Chronic kidney disease, Estimated glomerular filtration rate, Heart failure, Hypertension, Post-contrast acute kidney injury

## Abstract

**Purpose:**

To evaluate the associations between comorbidities and kidney function decline at 6-month and 1-year follow-up in outpatients with initial estimated glomerular filtration rate (eGFR) ≥ 30 mL/min/1.73 m^2^.

**Materials and methods:**

Outpatients aged 18 and older with confirmed diagnosis, who had eGFR ≥ 30 mL/min/1.73 m^2^ measured between April 2017 and March 2019, were included in this retrospective observational study. Of them, 30,595 included outpatients had 6-month eGFR test and 27,698 included outpatients had 1-year eGFR test. The outpatients were further divided into two groups based on initial eGFR: between 30 and 59 and ≥ 60 mL/min/1.73 m^2^. Impaired renal function was defined as eGFR declined to below 30 mL/min/1.73 m^2^. The comorbidities with P values less than 0.1 identified in univariable logistic regression models were entered into the multivariable analysis with backward selection, thereby identifying comorbidities that increased the risk of eGFR decline at 6-month and 1-year follow-up.

**Results:**

Outpatients with initial eGFR between 30 and 59 mL/min/1.73 m^2^ were 175.94 times more likely to have eGFR decline at 6 months, and were 94.10 times more likely to have eGFR decline at 1 year, compared with their corresponding initial eGFR ≥ 60 counterparts. Multivariable logistic regression analyses disclosed that chronic kidney disease, hypertension, and heart failure were independent risk factors for eGFR decline in outpatients with initial eGFR between 30 and 59 mL/min/1.73 m^2^.

**Conclusions:**

Outpatients with initial eGFR ≥ 60 mL/min/1.73 m^2^ might not need routine eGFR test prior to contrast-enhanced CT scan for 1 year. In addition, chronic kidney disease, hypertension, and heart failure increased the risk of declined renal function, particularly, in outpatients with initial eGFR between 30 and 59 mL/min/1.73 m^2^.

**Supplementary Information:**

The online version contains supplementary material available at 10.1007/s11604-023-01425-y.

## Introduction

Computed tomography (CT) using iodine-containing contrast media is a routine and highly informative diagnostic imaging technique. Most of the administered iodinated contrast media is excreted from the kidneys. However, post-contrast acute kidney injury (PC-AKI), one of the most serious adverse reactions to contrast media [1, 2], may occurred in some patients, particularly in patients with kidney disease [3–6]. PC-AKI often presents within 2 to 5 days after administration of contrast media, with an increase in the serum creatinine (sCr) level by 0.5 mg/dL or more (or 25% elevation or more) [1, 7]. The risk factors for PC-AKI may be procedure-related or patient-related [2]. And patient-related risk factors include aging, lower estimated glomerular filtration rate (eGFR), acute renal failure, chronic kidney disease (CKD), and congestive heart failure [2, 5, 8, 9].

In patients with eGFR less than 30 mL/min/1.73 m^2^, further deterioration of renal function may occur after contrast-enhanced CT scans [10]. A propensity score matching study concluded that the likelihood of PC-AKI increased by 51% in adult patients with pre-CT eGFR ≤ 30 mL/min/1.73 m^2^ [11]*.* Japanese guidelines recommend that patients with eGFR < 30 mL/min/1.73 m^2^ should not receive iodinated contrast media without appropriate precautions (e.g. using physiological saline intravenously) [5]. The European Society for Urogenital Radiology (ESUR) classifies three levels of renal dysfunction: eGFR < 60 mL/min/1.73 m^2^, eGFR < 45 mL/min/1.73 m^2^, and eGFR < 30 mL/min/1.73 m^2^; the last two levels are risk factors for PC-AKI [1, 2]. For patients with acute disease, patients with acute deterioration of chronic disease, or hospitalized patients, ESUR recommends evaluating renal function within 7 days before contrast media administration [1, 2]. In contrast, ESUR recommends that all other patients need to evaluate renal function within 3 months before contrast media administration [1, 2]. The American College of Radiology (ACR) also proposes that individual patient’s condition and associated risk factor(s) should be taken into consideration before using contrast media [3]. Therefore, in order to identify patients at high risk of PC-AKI, the measurement of eGFR, a hematologic marker of renal function, is suggested to be implemented before the administration of contrast media [1, 2, 5, 12, 13]. However, few studies have evaluated renal function over time, and the timing of eGFR test prior to contrast-enhanced CT has not been standardized.

The purpose of this retrospective observational study was to identify patient-related risk factors for impaired renal function at 6 months and 1 year. Thus, we retrospectively reviewed the variation in eGFR over time in outpatients, and selected outpatients whose eGFR values were decreased from ≥ 30 to < 30 mL/min/1.73 m^2^ at 6 months or 1 year after the initial eGFR evaluation. Univariable and multivariable logistic regression analyses were then performed to evaluate the associations between comorbidities and eGFR decline, thereby identifying independent risk factors for eGFR decline. The anticipated results of this study may provide insight into the timing of eGFR test prior to contrast-enhanced CT for patients with various comorbidities.

## Materials and Methods

### Study population

This retrospective observational study was reviewed and approved by the Institutional Review Board of our hospital (IRB approval number: 20–196). The requirement for signed informed consent was waived because of the retrospective nature of this study.

Outpatients who visited our hospital and underwent eGFR assessment, with or without contrast-enhanced CT, from April 2017 through March 2019 were initially included. Because the assessment of renal function differs between children and adults, outpatients younger than 18 years were excluded from the study. Regarding the selection of diseases of interest, we referred to guidelines for the use of contrast media [5] and chose diseases that are generally considered to cause renal dysfunction [1, 2, 14–19] or whose treatments are associated with renal dysfunction [20]. However, since most included outpatients had multiple comorbidities, all comorbidities found in included outpatients were documented in this study. The baseline disease characteristics of all included outpatient were extracted from medical records. And all included patients were outpatients at the baseline, 6-month follow-up, and 1-year follow-up.

### Kidney function evaluation

Outpatients’ diagnostic and renal function data were extracted from the clinical data warehouse of our hospital. Renal function was assessed based on the eGFR that was calculated using an equation specific for Japanese [21]. According to the classification of CKD [1, 2], outpatients were divided into three groups based on the initial eGFR: ≥ 60, between 30 and 59, and < 30 mL/min/1.73 m^2^. Outpatients with an initial eGFR of < 30 mL/min/1.73 m^2^ were excluded, because they already had impaired renal function and needed appropriate precaution for contrast-enhanced CT [1, 2, 5]. In this study, decline in renal function was defined as when eGFR dropped to below 30 mL/min/1.73 m^2^ at 6 months or 1 year after the initial evaluation.

### Statistical analysis

Age is presented as mean and standard deviation; the rest variables are presented as count and percentage. The differences between two initial eGFR groups were examined with the Fisher’s exact test, except for the difference in age that was examined with the independent two samples test. The association between initial eGFR value and eGFR decline (below 30 mL/min/1.73 m^2^) was evaluated by univariable logistic regression analysis. Subsequently, univariable logistic regression analyses were performed to evaluate the associations between comorbidity and eGFR decline within the initial eGFR group at 6 months and 1 year. The comorbidities with P values less than 0.1 identified in univariable logistic regression models were chosen to be entered into the multivariable analysis with backward selection. All statistical hypothesis tests were two-side, and the significance level was set as 0.05. The statistical analyses were performed using the IBM SPSS Statistics 25.0 (IBM Corporation, Armonk, NY, USA).

## Results

### Patient selection

**Fig. 1:**
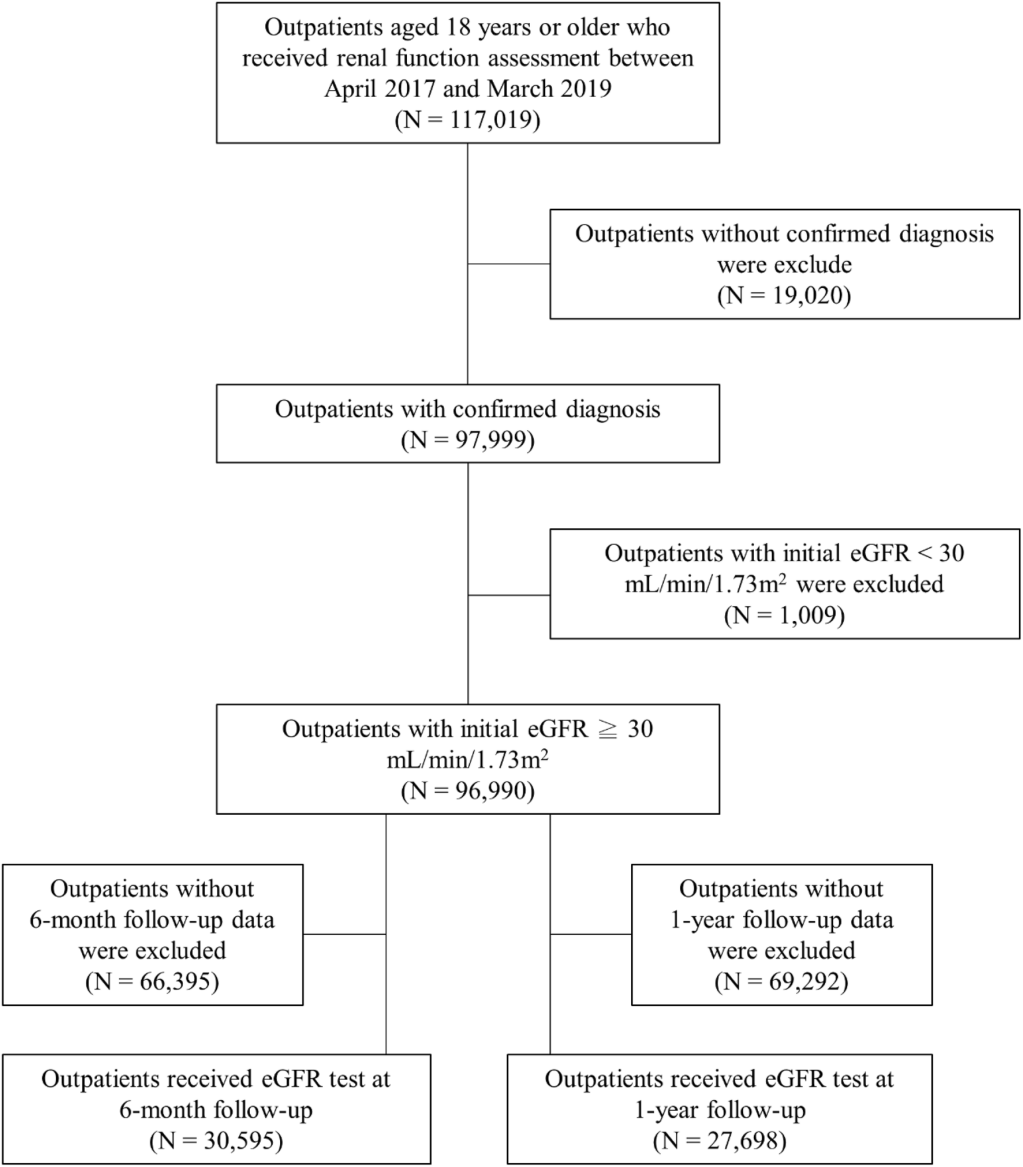
Flow diagram of patient selection

A total of 117,019 outpatients aged 18 years and older who underwent renal function test between April 2017 and March 2019 were initially selected for this retrospective study. The flowchart of outpatient selection is shown in Fig. 1. Of them, 19,020 outpatients who had no confirmed disease diagnosis and 1,009 outpatients who had initial eGFR < 30 mL/min/1.73m^2^ were excluded. As a result, 96,990 outpatients were included. After excluding 66,395 outpatients who did not have eGFR test at 6-month follow-up, the remaining 30,595 outpatients who had eGFR test at 6 months were subjected to the final analysis. In addition, after excluding 69,292 outpatients who did not have eGFR test at 1-year follow-up, the remaining 27,698 outpatients who had eGFR test at 1 year were independently analyzed in this retrospective study (Fig. 1).

### Baseline demographic and clinical characteristics

Among 30,595 outpatients with 6-month follow-up data, 5,686 outpatients had initial eGFR between 30 and 59 mL/min/1.73 m^2^, and 24,909 outpatients had initial eGFR ≥ 60 mL/min/1.73 m^2^ (Table 1). Outpatients with initial eGFR between 30 and 59 mL/min/1.73 m^2^ were significantly older than those with initial eGFR ≥ 60 mL/min/1.73 m^2^ (P < 0.001). There were significantly more males in the initial eGFR between 30 and 59 group compared with the initial eGFR ≥ 60 group (P < 0.001). Outpatients with initial eGFR between 30 and 59 mL/min/1.73 m^2^ had significant higher percentages of CKD, diabetes mellitus (DM), hypertension, acquired absence of kidney, glomerular diseases, gout or hyperuricemia, ischemic heart diseases, arrhythmia, heart failure, arteriosclerosis obliterans, hyperlipidemia, chronic obstructive pulmonary disease, cerebral infarction, gastric ulcer, IgG4-related disease, hyperthyroidism or hypothyroidism, migraine, asthma, malignant neoplasm of colon, and malignant neoplasms of urinary tract, than those with initial eGFR ≥ 60 mL/min/1.73 m^2^ (all p ≤ 0.03) (Table 1).

**Table 1:** Baseline demographic and clinical characteristics of the 30,595 outpatients who received eGFR test at 6 months after the initial eGFR measurement, stratified by initial eGFR value

	Initial eGFR between 30 and 59 (N = 5686)	Initial eGFR ≥ 60 (N = 24,909)	P value
Age (year)	71.8 (11.2)	60.0 (14.9)	< 0.001*
Gender			< 0.001*
Female	2,179 (38.3%)	13,280 (53.3%)	
Male	3,507 (61.7%)	11,629 (46.7%)	
Comorbidity			
Chronic kidney disease	803 (14.1%)	545 (2.2%)	< 0.001*
Diabetes mellitus	3,829 (67.3%)	12,986 (52.1%)	< 0.001*
Hypertension	3,541 (62.3%)	8,652 (34.7%)	< 0.001*
Acquired absence of kidney	11 (0.2%)	5 (0.0%)	< 0.001*
Glomerular diseases	800 (14.1%)	2,245 (9.0%)	< 0.001*
Gout or hyperuricemia	1,886 (33.2%)	3,065 (12.3%)	< 0.001*
Ischemic heart diseases	356 (6.3%)	665 (2.7%)	< 0.001*
Arrhythmia	1,831 (32.2%)	4,634 (18.6%)	< 0.001*
Heart failure	2,855 (50.2%)	6,894 (27.7%)	< 0.001*
Arteriosclerosis obliterans	774 (13.6%)	1,835 (7.4%)	< 0.001*
Hyperlipidemia	2,718 (47.8%)	8,353 (33.5%)	< 0.001*
Chronic obstructive pulmonary disease	255 (4.5%)	741 (3.0%)	< 0.001*
Cerebral infarction	1,215 (21.4%)	2,747 (11.0%)	< 0.001*
Gastric ulcer	3,274 (57.6%)	11,981 (48.1%)	< 0.001*
IgG4-related disease	608 (10.7%)	2,328 (9.3%)	0.002*
Schizophrenia or bipolar disorder	123 (2.2%)	614 (2.5%)	0.197
Hyperthyroidism or hypothyroidism	1,053 (18.5%)	3,739 (15.0%)	< 0.001*
Benign neoplasm of other and unspecified endocrine glands	47 (0.8%)	190 (0.8%)	0.620
Myasthenia gravis	31 (0.5%)	130 (0.5%)	0.847
Migraine	81 (1.4%)	581 (2.3%)	< 0.001*
Asthma	925 (16.3%)	3,764 (15.1%)	0.030*
Malignant neoplasm of colon	296 (5.2%)	963 (3.9%)	< 0.001*
Malignant neoplasm of bronchus and lung	395 (6.9%)	1,651 (6.6%)	0.394
Malignant neoplasm of urinary tract	313 (5.5%)	372 (1.5%)	< 0.001*
Parkinson’s disease	152 (2.7%)	597 (2.4%)	0.234

*P < 0.05 indicates a significant difference between the two groups

Among 27,698 outpatients with 1-year follow-up data, 4,954 outpatients had initial eGFR between 30 and 59 mL/min/1.73 m^2^, and 22,744 outpatients had initial eGFR ≥ 60 mL/min/1.73 m^2^ (Table 2). Outpatients with initial eGFR between 30 and 59 mL/min/1.73 m^2^ were significantly older than those with initial eGFR ≥ 60 mL/min/1.73 m^2^ (P < 0.001). There were significantly more males in the initial eGFR between 30 and 59 group compared with the initial eGFR ≥ 60 group (P < 0.001). Outpatients with initial eGFR between 30 and 59 mL/min/1.73 m^2^ had significant higher percentages of CKD, DM, hypertension, acquired absence of kidney, glomerular diseases, gout or hyperuricemia, ischemic heart diseases, arrhythmia, heart failure, arteriosclerosis obliterans, hyperlipidemia, chronic obstructive pulmonary disease, cerebral infarction, gastric ulcer, IgG4-related disease, hyperthyroidism or hypothyroidism, migraine, asthma, malignant neoplasm of colon, and malignant neoplasms of urinary tract, than those with initial eGFR ≥ 60 mL/min/1.73 m^2^ (all p ≤ 0.011) (Table 2).

**Table 2:** Baseline demographic and clinical characteristics of the 27,698 outpatients who received eGFR test at 1 year after the initial eGFR assessment, stratified by initial eGFR value

	Initial eGFR between 30 and 59 (N = 4954)	Initial eGFR ≥ 60 (N = 22,744)	P value
Age (year)	71.4 (11.1)	60.0 (14.7)	< 0.001*
Gender			< 0.001*
Female	1,880 (37.9%)	12,180 (53.6%)	
Male	3,074 (62.1%)	10,564 (46.4%)	
Comorbidity			
Chronic kidney disease	685 (13.8%)	517 (2.3%)	< 0.001*
Diabetes mellitus	3,350 (67.6%)	11,715 (51.5%)	< 0.001*
Hypertension	3,033 (61.2%)	7,534 (33.1%)	< 0.001*
Acquired absence of kidney	12 (0.2%)	6 (0.0%)	< 0.001*
Glomerular diseases	675 (13.6%)	1,986 (8.7%)	< 0.001*
Gout or hyperuricemia	1,635 (33.0%)	2,677 (11.8%)	< 0.001*
Ischemic heart diseases	297 (6.0%)	567 (2.5%)	< 0.001*
Arrhythmia	1,571 (31.7%)	4,228 (18.6%)	< 0.001*
Heart failure	2,433 (49.1%)	6,121 (26.9%)	< 0.001*
Arteriosclerosis obliterans	660 (13.3%)	1,586 (7.0%)	< 0.001*
Hyperlipidemia	2,336 (47.2%)	7,649 (33.6%)	< 0.001*
Chronic obstructive pulmonary disease	217 (4.4%)	670 (2.9%)	< 0.001*
Cerebral infarction	1,042 (21.0%)	2,465 (10.8%)	< 0.001*
Gastric ulcer	2,850 (57.5%)	10,855 (47.7%)	< 0.001*
IgG4-related disease	542 (10.9%)	2,051 (9.0%)	< 0.001*
Schizophrenia or bipolar disorder	97 (2.0%)	513 (2.3%)	0.220
Hyperthyroidism or hypothyroidism	953 (19.2%)	3,412 (15.0%)	< 0.001*
Benign neoplasm of other and unspecified endocrine glands	46 (0.9%)	170 (0.7%)	0.210
Myasthenia gravis	36 (0.7%)	116 (0.5%)	0.067
Migraine	72 (1.5%)	541 (2.4%)	< 0.001*
Asthma	815 (16.5%)	3,416 (15.0%)	0.011*
Malignant neoplasm of colon	252 (5.1%)	882 (3.9%)	< 0.001*
Malignant neoplasm of bronchus and lung	332 (6.7%)	1,456 (6.4%)	0.445
Malignant neoplasm of urinary tract	273 (5.5%)	331 (1.5%)	< 0.001*
Parkinson’s disease	121 (2.4%)	467 (2.1%)	0.090

*P < 0.05 indicates a significant difference between the two groups

### Association between initial eGFR and eGFR decline

In outpatients with 6-month follow-up data, 3.4% of outpatients with initial eGFR between 30 and 59 mL/min/1.73 m^2^ had eGFR decline, defined as eGFR decreasing to below 30 mL/min/1.73 m^2^, at 6 months. In contrast, only 0.02% of outpatients with initial eGFR ≥ 60 mL/min/1.73 m^2^ had eGFR decline at 6 months (Table 3). Univariable logistic regression analysis revealed that outpatients with initial eGFR between 30 and 59 mL/min/1.73 m^2^ were 175.94 times more likely to have eGFR decline at 6 months, compared to those with initial eGFR ≥ 60 mL/min/1.73 m^2^ (P < 0.001) (Table 3).

**Table 3:** Proportions of outpatients whose eGFR declined to below 30 mL/min/1.73 m^2^ at 6 months or 1 year after the initial eGFR assessment, and the associations between initial eGFR and eGFR decline

	eGFR declined to below 30	
	Count/N^†^ (percentage)	Odds ratio (95% CI)
Outpatients with 6-month follow-up (N = 30,595)		
Initial eGFR between 30 and 59	194/5,686 (3.4%)	175.94 (72.38, 427.68)
Initial eGFR ≥ 60	5/24,909 (0.02%)	Reference group
P value		< 0.001*
Outpatients with 1-year follow-up (N = 27,698)		
Initial eGFR between 30 and 59	253/4,954 (5.1%)	94.10 (53.85, 164.46)
Initial eGFR ≥ 60	13/22,744 (0.06%)	Reference group
P value		< 0.001*

^†^Count/N: the number of outpatients with eGFR decline over the total number of outpatients within the initial eGFR group

*P < 0.05 indicates a significant association between initial eGFR and the occurrence of eGFR decline

In outpatients with 1-year follow-up data, 5.1% of outpatients with initial eGFR between 30 and 59 mL/min/1.73 m^2^ had eGFR decline at 1 year. In contrast, only 0.06% of outpatients with initial eGFR ≥ 60 mL/min/1.73 m^2^ had eGFR decline at 1 year (Table 3). Univariable logistic regression analysis indicated that outpatients with initial eGFR between 30 and 59 mL/min/1.73 m^2^ were 94.10 times more likely to have eGFR decline at 1 year, compared to those with initial eGFR ≥ 60 mL/min/1.73 m^2^ (P < 0.001) (Table 3).

### Associations between comorbidities and eGFR decline in outpatients with initial eGFR between 30 and 59 mL/min/1.73 m^2^

Univariable logistic regression analysis revealed that in outpatients with initial eGFR between 30 and 59 mL/min/1.73 m^2^, CKD, DM, hypertension, glomerular diseases, gout or hyperuricemia, arrhythmia, and heart failure were significantly associated with eGFR decline at 6-month follow-up (all p ≤ 0.011) (Table 4). Moreover, in outpatients with initial eGFR between 30 and 59 mL/min/1.73 m^2^, CKD, DM, hypertension, glomerular diseases, gout or hyperuricemia, arrhythmia, heart failure, cerebral infarction, migraine, and malignant neoplasm of urinary tract were significantly associated with eGFR decline at 1-year follow-up (all p ≤ 0.024) (Table 5).

**Table 4:** The associations between comorbidities and eGFR decline at 6 month follow-up in outpatients with initial eGFR between 30 and 59 mL/min/1.73 m^2^ (N = 5686), determined by univariable logistic regression analysis

Status	Count/N^†^ (%)	Odds ratio (95% CI)	P value
Chronic kidney disease			
Yes	74/803 (9.22%)	4.03 (2.98, 5.44)	< 0.001*
No^#^	120/4883 (2.46%)		
Diabetes mellitus			
Yes	147/3829 (3.84%)	1.54 (1.10, 2.15)	0.011*
No^#^	47/1857 (2.53%)		
Hypertension			
Yes	157/3541 (4.43%)	2.64 (1.84, 3.80)	< 0.001*
No^#^	37/2145 (1.72%)		
Acquired absence of kidney			
Yes	0/11 (0.00%)	NA	
No^#^	194/5675 (3.42%)		
Glomerular diseases			
Yes	45/800 (5.63%)	1.89 (1.35, 2.67)	< 0.001*
No^#^	149/4886 (3.05%)		
Gout or hyperuricemia			
Yes	98/1886 (5.20%)	2.11 (1.59, 2.82)	< 0.001*
No^#^	96/3800 (2.53%)		
Ischemic heart diseases			
Yes	14/356 (3.93%)	1.17 (0.67, 2.04)	0.577
No^#^	180/5330 (3.38%)		
Arrhythmia			
Yes	81/1831 (4.42%)	1.53 (1.15, 2.05)	0.004*
No^#^	113/3855 (2.93%)		
Heart failure			
Yes	123/2855 (4.31%)	1.75 (1.30, 2.36)	< 0.001*
No^#^	71/2831 (2.51%)		
Arteriosclerosis obliterans			
Yes	35/774 (4.52%)	1.42 (0.97, 2.06)	0.068
No^#^	159/4912 (3.24%)		
Hyperlipidemia			
Yes	103/2718 (3.79%)	1.25 (0.93, 1.66)	0.134
No^#^	91/2968 (3.07%)		
Chronic obstructive pulmonary disease			
Yes	7/255 (2.75%)	0.79 (0.37, 1.70)	0.549
No^#^	187/5431 (3.44%)		
Cerebral infarction			
Yes	51/1215 (4.20%)	1.33 (0.96, 1.84)	0.090
No^#^	143/4471 (3.20%)		
Gastric ulcer			
Yes	119/3274 (3.63%)	1.18 (0.88, 1.58)	0.281
No^#^	75/2412 (3.11%)		
IgG4-related disease			
Yes	18/608 (2.96%)	0.85 (0.52, 1.39)	0.517
No^#^	176/5078 (3.47%)		
Schizophrenia or bipolar disorder			
Yes	2/123 (1.63%)	0.46 (0.11, 1.88)	0.282
No^#^	192/5563 (3.45%)		
Hyperthyroidism or hypothyroidism			
Yes	35/1053 (3.32%)	0.97 (0.67, 1.40)	0.862
No^#^	159/4633 (3.43%)		
Benign neoplasm of other and unspecified endocrine glands			
Yes	1/47 (2.13%)	0.61 (0.08, 4.47)	0.630
No^#^	193/5639 (3.42%)		
Myasthenia gravis			
Yes	1/31 (3.23%)	0.94 (0.13, 6.95)	0.954
No^#^	193/5655 (3.41%)		
Migraine			
Yes	0/81 (0.00%)	NA	
No^#^	194/5605 (3.46%)		
Asthma			
Yes	39/925 (4.22%)	1.31 (0.91, 1.87)	0.142
No^#^	155/4761 (3.26%)		
Malignant neoplasm of colon			
Yes	14/296 (4.73%)	1.44 (0.82, 2.51)	0.202
No^#^	180/5390 (3.34%)		
Malignant neoplasm of bronchus and lung			
Yes	10/395 (2.53%)	0.72 (0.38, 1.37)	0.320
No^#^	184/5291 (3.48%)		
Malignant neoplasm of urinary tract			
Yes	16/313 (5.11%)	1.57 (0.93, 2.66)	0.091
No^#^	178/5373 (3.31%)		
Parkinson’s disease			
Yes	7/152 (4.61%)	1.38 (0.64, 2.99)	0.413
No^#^	187/5534 (3.38%)		

NA: the odds ratio was not available due to the zero count

^†^Count/N: for status indicating “yes”, the number of outpatients with eGFR decline over the total number of outpatients with a particular comorbidity; for status indicating “no,” the number of outpatients with eGFR decline over the total number of outpatients without a particular comorbidity

^#^No: this group of outpatients served as the reference group for univariable regression analysis

*P < 0.05 indicates a significant association

**Table 5:** The associations between comorbidities and eGFR decline at 1-year follow-up in outpatients with initial eGFR between 30 and 59 mL/min/1.73 m^2^ (N = 4954), determined by univariable logistic regression analysis

Status	Count/N^†^ (%)	Odds ratio (95% CI)	P value
Chronic kidney disease			
Yes	113/685 (16.50%)	5.83 (4.48, 7.58)	< 0.001*
No^#^	140/4269 (3.28%)		
Diabetes mellitus			
Yes	189/3350 (5.64%)	1.44 (1.08, 1.92)	0.014*
No^#^	64/1604 (3.99%)		
Hypertension			
Yes	205/3033 (6.76%)	2.83 (2.05, 3.89)	< 0.001*
No^#^	48/1921 (2.50%)		
Acquired absence of kidney			
Yes	1/12 (8.33%)	0.59 (0.08, 4.60)	0.615
No^#^	252/4942 (5.10%)		
Glomerular diseases			
Yes	67/675 (9.93%)	2.42 (1.81, 3.25)	< 0.001*
No^#^	186/4279 (4.35%)		
Gout or hyperuricemia			
Yes	143/1635 (8.75%)	2.80 (2.16, 3.61)	< 0.001*
No^#^	110/3319 (3.31%)		
Ischemic heart diseases			
Yes	17/297 (5.72%)	1.14 (0.69, 1.89)	0.619
No^#^	236/4657 (5.07%)		
Arrhythmia			
Yes	94/1571 (5.98%)	1.29 (0.99, 1.68)	0.057
No^#^	159/3383 (4.70%)		
Heart failure			
Yes	157/2433 (6.45%)	1.74 (1.34, 2.26)	< 0.001*
No^#^	96/2521 (3.81%)		
Arteriosclerosis obliterans			
Yes	49/660 (7.42%)	1.61 (1.16, 2.22)	0.004*
No^#^	204/4294 (4.75%)		
Hyperlipidemia			
Yes	129/2336 (5.52%)	1.18 (0.91, 1.51)	0.210
No^#^	124/2618 (4.74%)		
Chronic obstructive pulmonary disease			
Yes	14/217 (6.45%)	1.30 (0.74, 2.27)	0.359
No^#^	239/4737 (5.05%)		
Cerebral infarction			
Yes	68/1042 (6.53%)	1.41 (1.06, 1.87)	0.020*
No^#^	185/3912 (4.73%)		
Gastric ulcer			
Yes	151/2850 (5.30%)	1.10 (0.85, 1.42)	0.477
No^#^	102/2104 (4.85%)		
IgG4-related disease			
Yes	28/542 (5.17%)	1.01 (0.68, 1.52)	0.947
No^#^	225/4412 (5.10%)		
Schizophrenia or bipolar disorder			
Yes	6/97 (6.19%)	1.23 (0.53, 2.84)	0.627
No^#^	247/4857 (5.09%)		
Hyperthyroidism or hypothyroidism			
Yes	58/953 (6.09%)	1.26 (0.94, 1.71)	0.127
No^#^	195/4001 (4.87%)		
Benign neoplasm of other and unspecified endocrine glands			
Yes	0/46 (0.00%)	NA	
No^#^	253/4908 (5.15%)		
Myasthenia gravis			
Yes	1/36 (2.78%)	0.53 (0.07, 3.88)	0.531
No^#^	252/4918 (5.12%)		
Migraine			
Yes	8/72 (11.11%)	2.37 (1.12, 4.99)	0.024*
No	245/4882 (5.02%)		
Asthma			
Yes	38/815 (4.66%)	0.89 (0.63, 1.27)	0.529
No^#^	215/4139 (5.19%)		
Malignant neoplasm of colon			
Yes	17/252 (6.75%)	1.37 (0.82, 2.28)	0.227
No^#^	236/4702 (5.02%)		
Malignant neoplasm of bronchus and lung			
Yes	10/332 (3.01%)	0.56 (0.29, 1.06)	0.077
No^#^	243/4622 (5.26%)		
Malignant neoplasm of urinary tract			
Yes	24/273 (8.79%)	1.87 (1.21, 2.91)	0.005*
No^#^	229/4681 (4.89%)		
Parkinson’s disease			
Yes	6/121 (4.96%)	0.97 (0.42, 2.22)	0.940
No^#^	247/4833 (5.11%)		

^†^Count/N: for status indicating “yes”, the number of outpatients with eGFR decline over the total number of outpatients with a particular comorbidity; for status indicating “no,” the number of outpatients with eGFR decline over the total number of outpatients without a particular comorbidity

NA: the odds ratio was not available due to the zero count

^#^No: this group of outpatients served as the reference group for univariable regression analysis

*P < 0.05 indicates a significant association

### Associations between comorbidities and eGFR decline in outpatients with initial eGFR ≥ 60 mL/min/1.73 m^2^

Univariable logistic regression analysis disclosed that in outpatients with initial eGFR ≥ 60 mL/min/1.73 m^2^, CKD and heart failure were significantly associated with eGFR decline at 6-month follow-up (both p ≤ 0.036) (Supplementary Table 1). Furthermore, in outpatients with initial eGFR ≥ 60 mL/min/1.73 m^2^, hypertension, gout or hyperuricemia, ischemic heart diseases, arrhythmia, heart failure, and myasthenia gravis were significantly associated with eGFR decline at 1-year follow-up (all p ≤ 0.045) (Supplementary Table 2).

### Independent risk factors for eGFR decline identified by multivariable logistic regression analysis

To investigate whether the comorbidities had independent and significant influence on the risk of eGFR declined to below 30, four multivariable logistic regression models were conducted. The comorbidities with P values less than 0.1 in Tables 4, 5 and Supplementary Tables 1, 2 were entered into the process of model selection for the corresponding multivariable logistic regression analysis.

Multivariable logistic regression analysis revealed that in outpatients with initial eGFR between 30 and 59 mL/min/1.73 m^2^, CKD, hypertension, and heart failure were significant risk factors for eGFR decline at 6-month follow-up (all p ≤ 0.034) (Table 6). However, more comorbidities, including CKD, hypertension, glomerular diseases, gout or hyperuricemia, heart failure, migraine, and malignant neoplasm of urinary tract, were significant risk factors for eGFR decline at 1-year follow-up in outpatients with initial eGFR between 30 and 59 mL/min/1.73 m^2^ (all p ≤ 0.014) (Table 6).

**Table 6:** Summary of independent risk factors for eGFR decline identified by multivariable logistic regression analysis

	Adjusted odds ratio (95% CI)	P value
Initial eGFR between 30 and 59 mL/min/1.73 m^2^		
Outpatients evaluated at 6 months (N = 5686)		
Chronic kidney disease	3.18 (2.33, 4.36)	< 0.001*
Hypertension	1.79 (1.21, 2.63)	0.003*
Glomerular diseases	1.41 (0.98, 2.01)	0.061
Gout or hyperuricemia	1.35 (0.99, 1.84)	0.056
Heart failure	1.41 (1.03, 1.93)	0.034*
Malignant neoplasm of urinary tract	1.62 (0.95, 2.76)	0.079
Outpatients evaluated at 1 year (N = 4954)		
Chronic kidney disease	4.33 (3.28, 5.72)	< 0.001*
Hypertension	1.75 (1.24, 2.46)	0.002*
Glomerular diseases	1.63 (1.19, 2.22)	0.002*
Gout or hyperuricemia	1.63 (1.24, 2.16)	< 0.001*
Heart failure	1.42 (1.07, 1.88)	0.014*
Migraine	2.73 (1.25, 5.98)	0.012*
Malignant neoplasm of urinary tract	1.88 (1.18, 2.98)	0.008*
Initial eGFR ≥ 60 mL/min/1.73 m^2^		
Outpatients evaluated at 6 months (N = 24,909)		
Chronic kidney disease	23.60 (3.90, 142.84)	< 0.001*
Heart failure	8.85 (0.98, 79.97)	0.052
Outpatients evaluated at 1 year (N = 22,744)		
Hypertension	5.62 (1.51, 20.95)	0.010*
Arrhythmia	2.57 (0.85, 7.83)	0.096
Heart failure	16.54 (2.11, 129.64)	0.008*

*P < 0.05 indicated significant association

On the other hand, multivariable logistic regression analysis disclosed that in outpatients with initial eGFR ≥ 60 mL/min/1.73 m^2^, only CKD was significant risk factor for eGFR decline at 6-month follow-up (P < 0.001) (Table 6). Moreover, hypertension and heart failure significant risk factors for eGFR decline at 1-year follow-up in outpatients with initial eGFR ≥ 60 mL/min/1.73 m^2^ (both p ≤ 0.010) (Table 6).

### Associations between contrast administration and eGFR decline in outpatients with initial eGFR between 30 and 59 mL/min/1.73 m^2^

As revealed in Table 3, outpatients with initial eGFR between 30 and 59 mL/min/1.73 m^2^ were at significantly increased risk of eGFR decline at 6 months and 1 year (OR = 175.94 and 94.10, respectively). So, the associations between contrast administration and eGFR decline, regardless of comorbidities, were assessed in outpatients with initial eGFR between 30 and 59 mL/min/1.73 m^2^. Logistic regression analysis revealed that contrast administration was not significantly associated with eGFR decline in outpatients receiving eGFR test at 6 months (OR = 1.03, P = 0.886) (Supplementary Table 3). And contrast administration was also not significantly associated with eGFR decline in outpatients receiving eGFR test at 1 year (OR = 0.75, P = 0.100) (Supplementary Table 3).

## Discussion

In the present retrospective observational study, 30,595 outpatients with eGFR test at 6 months and 27,698 outpatients with eGFR test at 1 year were independently analyzed. The results indicated that at both 6 months and 1 year, outpatients with initial eGFR between 30 and 59 mL/min/1.73 m^2^ were older and had higher percentages of comorbidities, than their corresponding initial eGFR ≥ 60 counterparts. Incidence rates of eGFR decline at 6 months and 1 year were 0.02% and 0.06%, respectively, in outpatients with initial eGFR ≥ 60 mL/min/1.73 m^2^, but were 3.4% and 5.1%, respectively, in outpatients with initial eGFR between 30 and 59 mL/min/1.73 m^2^. Outpatients with initial eGFR between 30 and 59 mL/min/1.73 m^2^ were 175.94 times more likely to have eGFR decline at 6 months, and were 94.10 times more likely to have eGFR decline at 1 year, compared with their corresponding initial eGFR ≥ 60 counterparts. Multivariable logistic regression analysis disclosed that in outpatients with initial eGFR between 30 and 59 mL/min/1.73 m^2^, CKD, hypertension, and heart failure were independent risk factors for eGFR decline at 6 months. In addition to the above-mentioned comorbidities, glomerular diseases, gout or hyperuricemia, migraine, and malignant neoplasm of urinary tract were also independent risk factors for eGFR decline at 1 year for outpatients with initial eGFR between 30 and 59 mL/min/1.73 m^2^. On the contrary, in outpatients with initial eGFR ≥ 60 mL/min/1.73 m^2^, only CKD was independent risk factor for eGFR decline at 6 months, and both hypertension and heart failure were independent risk factors for eGFR decline at 1 year.

Current findings suggested that the vast majority of outpatients with initial eGFR ≥ 60 mL/min/1.73 m^2^ still have sufficient renal function to receive contrast-enhanced CT scan at 6 month and 1 year, and that annual renal function reassessment prior to the use of contrast media may be suitable for such outpatients. In hospitalized children with pre-CT eGFR ≥ 60 mL/min/1.73 m^2^, iodinated contrast media was not associated with PC-AKI [22]. On the other hand, it is widely acknowledged that renal function declines with age [23, 24], and we also found outpatients with lower initial eGFR were older.

Outpatients with initial eGFR between 30 and 59 mL/min/1.73 m^2^ had higher incidence of almost all comorbidities documented in this study. Of them, outpatients with CKD, hypertension, or heart failure were more likely to have eGFR decline at 6 months and 1 year. Our findings were consistent with the results of previous studies [1, 2, 14–19], and a questionnaire survey study conducted in Japan, which concluded that chronic and acute kidney diseases (96.7% and 93.6%, respectively) were common risk factors for PC-AKI in Japan [25].

Age, sex, and sCr are considered in the current formula-based calculation of eGFR [26]. However, the concentration of sCr is altered by diurnal variation, menstrual cycle, nutritional status, and muscle mass to some degree [27]. A recent meta-analysis evaluated the influence of the within-subject biological variation of sCr on the reliability of eGFR, and concluded that eGFR can discriminate between true change in kidney function and random fluctuation [28]. Thus, despite biological variation of sCr, eGFR is a reliable tool for the evaluation of kidney function [28, 29].

On the other hand, gadolinium (Gd)-based contrast media are often used for contrast-enhanced magnetic resonance imaging [30]*,* but are associated with tissue retention of Gd and nephrogenic systemic fibrosis [31, 32]. On top of that, contrast media extravasation may further worsen the impact of contrast media to patients [33]. Multiple lines of evidence suggested that patients with an eGFR of < 30 mL/min/1.73 m^2^ should not receive Gd-based contrast media without appropriate precautions [34, 35]. A questionnaire survey study revealed that in Japan, eGFR test was most frequently performed prior to the use of iodinated and Gd-based contrast media (80.8% and 82.6%, respectively) [25].

Several limitations of this study needed to be addressed. First of all, we extracted the disease diagnosis information according to the diagnoses entered into the database. In actual clinical practice, some diagnoses might reflect the wordings required by insurance companies; therefore, the possibility of outpatient misclassification cannot be ruled out. Second, although all included patients were outpatients at the baseline, 6-month follow-up, and 1-year follow-up, whether the included outpatients were hospitalized during the study period was not documented in this study. Third, the data analyzed were from a single institution, indeed a university hospital (an academic medical center). Generally speaking, patients admitted to a university hospital have more severe conditions than those admitted to general medical institutions. Hence, the incidence of renal function decline over the study period might be overestimated in this study, compared to the nationwide incidence. Fourth, most included outpatients had multiple comorbidities, and all comorbidities found in included outpatients were documented in this study. A huge variety of medicines were taken by the included outpatients. On top of that, dosage and frequency would further complicate the analysis of medicines, exceeding the word count limit. Further research is warranted to investigate the influence of medicines for CKD, hypertension, or heart failure on eGFR decline.

In conclusion, both renal function and comorbidity at the initial eGFR examination should be considered to deduce the appropriate timing regarding renal function evaluation before the use of contrast media. Outpatients with initial eGFR ≥ 60 mL/min/1.73 m^2^ were much less likely to have eGFR decline at 6 months and 1 year, compared with their corresponding initial eGFR between 30 and 59 counterparts. Therefore, outpatients with initial eGFR ≥ 60 mL/min/1.73 m^2^ might not need routine eGFR test prior to contrast-enhanced CT scan. Furthermore, CKD, hypertension, and heart failure were main risk factors for renal function decline. If outpatients were diagnosed with CKD, hypertension, or heart failure, close monitoring eGFR might be necessary, particularly, in outpatients with initial eGFR between 30 and 59 mL/min/1.73 m^2^.

## Supplementary Information

Below is the link to the electronic supplementary material.

Supplementary file1 (DOCX 33 KB)
