# Supplementary material for: Changes in renal function over time in outpatients with eGFR ≥ 30 mL/min/1.73 m2: implication for timing of renal function testing before contrast-enhanced CT imaging
Source: Jpn J Radiol. 2023 Apr 11;41(9):994–1006. doi: 10.1007/s11604-023-01425-y (PMC10469099; doi:10.1007/s11604-023-01425-y)
Supplement: Supplementary file 1 — Supplementary file1 (DOCX 33 KB) [file 11604_2023_1425_MOESM1_ESM.docx]

**Supplementary Table 1.** The associations between comorbidities and eGFR decline at 6-month follow-up in outpatients with initial eGFR ≥ 60 mL/min/1.73 m^2^ (N = 24909), determined by univariable logistic regression analysis.

| **Status** | | **Count/N**† **(%)** | |  | **Odds ratio (95% CI)** | | | **P-value** | |  |
| --- | --- | --- | --- | --- | --- | --- | --- | --- | --- | --- |
| Chronic kidney disease | Yes | | 2 / 545 (0.37%) | | |  | 29.91 (4.99, 179.36) | | <0.001* | |
|  | No^#^ | | 3 / 24,364 (0.01%) | | |  |  | |  | |
| Diabetes mellitus | Yes | | 3 / 12,986 (0.02%) | | |  | 1.38 (0.23, 8.24) | | 0.726 | |
|  | No^#^ | | 2 / 11,923 (0.02%) | | |  |  | |  | |
| Hypertension | Yes | | 5 / 8,652 (0.06%) | | |  | NA | |  | |
|  | No^#^ | | 0 / 16,257 (0.00%) | | |  |  |  |  | |
| Acquired absence of kidney | Yes | | 0 / 5 (0.00%) | | |  | NA | |  | |
|  | No^#^ | | 5 / 24,904 (0.02%) | | |  |  |  |  | |
| Glomerular diseases | Yes | | 0 / 2,245 (0.00%) | | |  | NA | |  | |
|  | No^#^ | | 5 / 22,664 (0.02%) | | |  |  |  |  | |
| Gout or hyperuricemia | Yes | | 2 / 3,065 (0.07%) | | |  | 4.75 (0.79, 28.46) | | 0.088 | |
|  | No^#^ | | 3 / 21,844 (0.01%) | | |  |  | |  | |
| Ischemic heart diseases | Yes | | 0 / 665 (0.00%) | | |  | NA | |  | |
|  | No^#^ | | 5 / 24,244 (0.02%) | | |  |  |  |  | |
| Arrhythmia | Yes | | 1 / 4,634 (0.02%) | | |  | 1.09 (0.12, 9.79) | | 0.936 | |
|  | No^#^ | | 4 / 20,275 (0.02%) | | |  |  | |  | |
| Heart failure | Yes | | 4 / 6,894 (0.06%) | | |  | 10.46 (1.17, 93.58) | | 0.036* | |
|  | No^#^ | | 1 / 18,015 (0.01%) | | |  |  | |  | |
| Arteriosclerosis obliterans | Yes | | 1 / 1,835 (0.05%) | | |  | 3.14 (0.35, 28.15) | | 0.306 | |
|  | No^#^ | | 4 / 23,074 (0.02%) | | |  |  | |  | |
| Hyperlipidemia | Yes | | 2 / 8,353 (0.02%) | | |  | 1.32 (0.22, 7.91) | | 0.760 | |
|  | No^#^ | | 3 / 16,556 (0.02%) | | |  |  | |  | |
| Chronic obstructive pulmonary disease | Yes | | 0 / 741 (0.00%) | | |  | NA | |  | |
|  | No^#^ | | 5 / 24,168 (0.02%) | | |  |  |  |  | |
| Cerebral infarction | Yes | | 0 / 2,747 (0.00%) | | |  | NA | |  | |
|  | No^#^ | | 5 / 22,162 (0.02%) | | |  |  |  |  | |
| Gastric ulcer | Yes | | 5 / 11,981 (0.04%) | | |  | NA | |  | |
|  | No^#^ | | 0 / 12,928 (0.00%) | | |  |  |  |  | |
| IgG4-related disease | Yes | | 0 / 2,328 (0.00%) | | |  | NA | |  | |
|  | No^#^ | | 5 / 22,581 (0.02%) | | |  |  |  |  | |
| Schizophrenia or bipolar disorder | Yes | | 0 / 614 (0.00%) | | |  | NA | |  | |
|  | No^#^ | | 5 / 24,295 (0.02%) | | |  |  |  |  | |
| Hyperthyroidism or hypothyroidism | Yes | | 2 / 3,739 (0.05%) | | |  | 3.78 (0.63, 22.61) | | 0.146 | |
|  | No^#^ | | 3 / 21,170 (0.01%) | | |  |  | |  | |
| Benign neoplasm of other and unspecified endocrine glands | Yes | | 0 / 190 (0.00%) | | |  | NA | |  | |
|  | No^#^ | | 5 / 24,719 (0.02%) | | |  |  |  |  | |
| Myasthenia gravis | Yes | | 0 / 130 (0.00%) | | |  | NA | |  | |
|  | No^#^ | | 5 / 24,779 (0.02%) | | |  |  |  |  | |
| Migraine | Yes | | 0 / 581 (0.00%) | | |  | NA | |  | |
|  | No^#^ | | 5 / 24,328 (0.02%) | | |  |  |  |  | |
| Asthma | Yes | | 1 / 3,764 (0.03%) | | |  | 1.40 (0.16, 12.57) | | 0.761 | |
|  | No^#^ | | 4 / 21,145 (0.02%) | | |  |  | |  | |
| Malignant neoplasm of colon | Yes | | 0 / 963 (0.00%) | | |  | NA | |  | |
|  | No^#^ | | 5 / 23,946 (0.02%) | | |  |  |  |  | |
| Malignant neoplasm of bronchus  and lung | Yes | | 1 / 1,651 (0.06%) | | |  | 3.52 (0.39, 31.54) | | 0.260 | |
|  | No^#^ | | 4 / 23,258 (0.02%) | | |  |  | |  | |
| Malignant neoplasm of urinary  tract | Yes | | 0 / 372 (0.00%) | | |  | NA | |  | |
|  | No^#^ | | 5 / 24,537 (0.02%) | | |  |  |  |  | |
| Parkinson’s disease | Yes | | 0 / 597 (0.00%) | | |  | NA | |  | |
|  | No^#^ | | 5 / 24,312 (0.02%) | | |  |  |  |  | |

†Count/N: for status indicating “yes”, the number of outpatients with eGFR decline over the total number of outpatients with a particular comorbidity; for status indicating “no,” the number of outpatients with eGFR decline over the total number of outpatients without a particular comorbidity.

NA: the odds ratio was not available due to the zero count.

^#^No: this group of outpatients served as the reference group for univariable regression analysis.

*P<0.05 indicates a significant association.

**Supplementary Table 2.** The associations between comorbidities and eGFR decline at 1-year follow-up in outpatients with initial eGFR ≥ 60 mL/min/1.73 m^2^ (N = 22744), determined by univariable logistic regression analysis.

| **Status** | | **Count**†**/N (%)** | | **Odds ratio (95% CI)** | | **P-value** | |
| --- | --- | --- | --- | --- | --- | --- | --- |
| Chronic kidney disease | Yes | | 1 / 517 (0.19%) | | 3.59 (0.47, 27.64) | | 0.220 |
|  | No^#^ | | 12 / 22,227 (0.05%) | |  | |  |
| Diabetes mellitus | Yes | | 7 / 11,715 (0.06%) | | 1.10 (0.37, 3.27) | | 0.866 |
|  | No^#^ | | 6 / 11,029 (0.05%) | |  | |  |
| Hypertension | Yes | | 10 / 7,534 (0.13%) | | 6.74 (1.85, 24.49) | | 0.004* |
|  | No^#^ | | 3 / 15,210 (0.02%) | |  | |  |
| Acquired absence of kidney | Yes | | 0 / 6 (0.00%) | | NA | |  |
|  | No^#^ | | 13 / 22,738 (0.06%) | |  |  |  |
| Glomerular diseases | Yes | | 1 / 1,986 (0.05%) | | 0.87 (0.11, 6.70) | | 0.894 |
|  | No^#^ | | 12 / 20,758 (0.06%) | |  | |  |
| Gout or hyperuricemia | Yes | | 4 / 2,677 (0.15%) | | 3.34 (1.03, 10.84) | | 0.045* |
|  | No^#^ | | 9 / 20,067 (0.04%) | |  | |  |
| Ischemic heart diseases | Yes | | 2 / 567 (0.35%) | | 7.13 (1.58, 32.26) | | 0.011* |
|  | No^#^ | | 11 / 22,177 (0.05%) | |  | |  |
| Arrhythmia | Yes | | 6 / 4,228 (0.14%) | | 3.76 (1.26, 11.19) | | 0.017* |
|  | No^#^ | | 7 / 18,516 (0.04%) | |  | |  |
| Heart failure | Yes | | 8 / 6,121 (0.13%) | | 4.35 (1.42, 13.30) | | 0.010* |
|  | No^#^ | | 5 / 16,623 (0.03%) | |  | |  |
| Arteriosclerosis obliterans | Yes | | 2 / 1,586 (0.13%) | | 2.43 (0.54, 10.96) | | 0.249 |
|  | No^#^ | | 11 / 21,158 (0.05%) | |  | |  |
| Hyperlipidemia | Yes | | 5 / 7,649 (0.07%) | | 1.23 (0.40, 3.77) | | 0.713 |
|  | No^#^ | | 8 / 15,095 (0.05%) | |  | |  |
| Chronic obstructive pulmonary disease | Yes | | 0 / 670 (0.00%) | | NA | |  |
|  | No^#^ | | 13 / 22,074 (0.06%) | |  |  |  |
| Cerebral infarction | Yes | | 1 / 2,465 (0.04%) | | 0.69 (0.09, 5.27) | | 0.717 |
|  | No^#^ | | 12 / 20,279 (0.06%) | |  | |  |
| Gastric ulcer | Yes | | 7 / 10,855 (0.06%) | | 1.28 (0.43, 3.80) | | 0.659 |
|  | No^#^ | | 6 / 11,889 (0.05%) | |  | |  |
| IgG4-related disease | Yes | | 2 / 2,051 (0.10%) | | 1.84 (0.41, 8.29) | | 0.430 |
|  | No^#^ | | 11 / 20,693 (0.05%) | |  | |  |
| Schizophrenia or bipolar disorder | Yes | | 1 / 513 (0.19%) | | 3.62 (0.47, 27.86) | | 0.217 |
|  | No^#^ | | 12 / 22,231 (0.05%) | |  | |  |
| Hyperthyroidism or hypothyroidism | Yes | | 2 / 3,412 (0.06%) | | 1.03 (0.23, 4.65) | | 0.969 |
|  | No^#^ | | 11 / 19,332 (0.06%) | |  | |  |
| Benign neoplasm of other and unspecified endocrine glands | Yes | | 0 / 170 (0.00%) | | NA | |  |
|  | No^#^ | | 13 / 22,574 (0.06%) | |  |  |  |
| Myasthenia gravis | Yes | | 1 / 116 (0.86%) | | 16.39 (2.11, 127.08) | | 0.007* |
|  | No^#^ | | 12 / 22,628 (0.05%) | |  | |  |
| Migraine | Yes | | 0 / 541 (0.00%) | | NA | |  |
|  | No^#^ | | 13 / 22,203 (0.06%) | |  |  |  |
| Asthma | Yes | | 2 / 3,416 (0.06%) | | 1.03 (0.23, 4.64) | | 0.971 |
|  | No^#^ | | 11 / 19,328 (0.06%) | |  | |  |
| Malignant neoplasm of colon | Yes | | 1 / 882 (0.11%) | | 2.07 (0.27, 15.91) | | 0.486 |
|  | No^#^ | | 12 / 21,862 (0.05%) | |  | |  |
| Malignant neoplasm of bronchus  and lung | Yes | | 0 / 1,456 (0.00%) | | NA | |  |
|  | No^#^ | | 13 / 21,288 (0.06%) | |  |  |  |
| Malignant neoplasm of urinary tract | Yes | | 1 / 331 (0.30%) | | 5.66 (0.73, 43.63) | | 0.096 |
|  | No | | 12 / 22,413 (0.05%) | |  | |  |
| Parkinson’s disease | Yes | | 0 / 467 (0.00%) | | NA | |  |
|  | No^##^ | | 13 / 22,277 (0.06%) | |  |  |  |

†Count/N: for status indicating “yes”, the number of outpatients with eGFR decline over the total number of outpatients with a particular comorbidity; for status indicating “no,” the number of outpatients with eGFR decline over the total number of outpatients without a particular comorbidity.

NA: the odds ratio was not available due to zero count.

#No: this group of outpatients served as the reference group for univariable regression analysis.

*P<0.05 indicates a significant association.

**Supplementary Table 3.** The associations between contrast administration and eGFR decline in outpatients with initial eGFR between

30–59, stratified by follow-up.

|  | | Administration of  iodine-containing contrast media | eGFR declined to below 30 | | |
| --- | --- | --- | --- | --- | --- |
|  |  |  | Count / N (percentage) | Odds ratio (95% CI) | P-value |
| Outpatients with 6-month follow-up (N = 5,686) |  | Yes | 37/1062 (3.48%) | 1.03 (0.71, 1.48) | 0.886 |
|  |  | No | 157/4624 (3.40%) | reference group |  |
| Outpatients with 1-year follow-up (N = 4,954) |  | Yes | 39/962 (4.05%) | 0.75 (0.53, 1.06) | 0.100 |
|  |  | No | 214/3992 (5.36%) | reference group |  |
